# Participation in a Community-Based Women's Health Education Program and At-Risk Child Development in Rural Kenya: Developmental Screening Questionnaire Results Analysis

**DOI:** 10.9745/GHSP-D-20-00349

**Published:** 2021-12-31

**Authors:** Megan S. McHenry, Lauren Y. Maldonado, Ziyi Yang, Gertrude Anusu, Evelyn Kaluhi, Astrid Christoffersen-Deb, Julia J. Songok, Laura J. Ruhl

**Affiliations:** a Academic Model Providing Access to Healthcare (AMPATH), Eldoret, Kenya.; b Indiana University School of Medicine, Indianapolis, IN, USA.; c Massachusetts General Hospital, Departments of Medicine and Pediatrics, Boston, MA, USA.; d University of British Columbia, Department of Obstetrics and Gynecology, Vancouver, Canada.; e University of Toronto, Department of Obstetrics and Gynecology, Toronto, Canada.; f Moi University College of Health Sciences, Department of Child Health and Paediatrics, Eldoret, Kenya.

## Abstract

**Background:**

Over 43% of children living in low- and middle-income countries are at risk for developmental delays; however, access to protective interventions in these settings is limited. We evaluated the effect of maternal participation in Chamas for Change (Chamas)—a community-based women's health education program during pregnancy and postpartum—and risk of developmental delay among their children in rural Kenya.

**Methods:**

We analyzed developmental screening questionnaire (DSQ) data from a cluster randomized controlled trial in Trans Nzoia County, Kenya (ClinicalTrials.gov, NCT03187873). Intervention clusters (Chamas) participated in community health volunteer-led, group-based health lessons twice a month during pregnancy and postpartum; controls had monthly home visits (standard of care). We screened all children born during the trial who were alive at 1-year follow-up. We labeled children with any positive item on the DSQ as “at-risk development.” We analyzed data using descriptive statistics and multilevel regression models (α=.05); analyses were intention-to-treat using individual-level data.

**Results:**

Between November 2017 and March 2018, we enrolled 1,920 pregnant women to participate in the parent trial. At 1-year follow-up, we screened 1,273 (689 intervention, 584 control) children born during the trial with the DSQ. Intervention mothers had lower education levels and higher poverty likelihood scores than controls (*P*<.001 and *P*=.007, respectively). The overall rate of at-risk development was 3.5%. Children in Chamas clusters demonstrated significantly lower rates of at-risk development than controls (2.5% vs. 4.8%, *P*=.025). Adjusted analyses revealed lower odds for at-risk development in the intervention arm (OR=0.50; 95% confidence interval=0.27, 0.94).

**Conclusions:**

Maternal participation in a community-based women's health education program was associated with lower rates of at-risk development compared to the standard of care. Overall, rates of at-risk development were lower than expected for this population, warranting further investigation. Chamas may help protect children from developmental delay in rural Kenya and other resource-limited settings.

## Abstract (precis)

A community-based intervention focused on women's health education may help protect against early childhood developmental delays in resource-limited settings.

## Teaser

**Key Findings:**

- Infants in Chamas had a lower likelihood of at-risk development compared to those in the control group. Older infant age at time of screening and having natural or uncovered walls was also associated with lower odds of at-risk development.
- Our findings offer evidence to support that by addressing health literacy, providing community in a supportive environment, and offering a means to combat social determinants of child developmental delays, the Chamas for Change intervention may help protect children from developmental delays in this resource-limited setting in Kenya.

**Key Implications:**

- When looking to revise current strategies to help protect children from developmental delay in this setting, policy makers should consider interventions that leverage existing resources to ensure their sustainability.

## INTRODUCTION

Early childhood development (ECD) lays the foundation upon which every individual's cognitive, social, and emotional abilities are built. As such, investing in ECD not only benefits individuals but also boosts shared prosperity and helps eliminate extreme poverty.^1^^,^^2^ However, recent data suggest that more than 43% of children under age 5 years living in low- and middle-income countries (LMICs) are at risk for developmental delays.^3^ Among children at risk, more than 60% reside in sub-Saharan Africa.^4^ This inequity perpetuates intergenerational cycles of poverty, particularly in rural communities where children are often denied equal access to resources and opportunities that nurture ECD.

The World Health Organization (WHO) estimates that 45% of children under age 5 years in Kenya are at risk for developmental delays (56% rural, 25% urban), translating to an estimated 138% loss in annual adult wages.^5^ Multifaceted challenges have limited the success of recent efforts (e.g., government policies, school-based programming) to bolster ECD. These challenges include inadequate financial investment in early education, poor access to health services that protect against known risk factors (e.g., malnutrition, recurrent and/or chronic infections), and limited awareness of the deleterious role of chronic stress (e.g., harsh punishment) on development.^6^ Moreover, few policies focused on ECD target the first 1,000 days of life—a critical period during which the brain undergoes rapid change and establishes a framework for lifelong learning.^7^ Inadequate antenatal care,^8^^–^^10^ malnutrition,^1^ and maternal depression,^11^ during this period can also have long-standing negative consequences.^1^^,^^7^^,^^12^ Effective interventions that protect against developmental delays within rural, resource-limited settings are urgently needed to address these disparities.

In 2012, the Academic Model Providing Access to Healthcare partnership and Republic of Kenya Ministry of Health established the Chamas for Change (Chamas) program to help improve maternal, newborn, and child health (MNCH) in rural western Kenya.^13^^,^^14^ Chamas is a longitudinal program that supports women during pregnancy and for the first 1,000 days of the infants' lives with community-driven, group-based health education. Importantly, this intervention integrates known strategies to improve outcomes for women and infants, including ECD, while leveraging existing resources in rural Kenyan communities. A pilot study evaluating the first year of this program demonstrated significant associations between participation and the likelihood of practicing positive MNCH behaviors, including attending adequate antenatal care and exclusively breastfeeding.^14^^,^^15^

A pilot study evaluating the first year of Chamas demonstrated significant associations between participation and the likelihood of practicing positive MNCH behaviors, including attending adequate antenatal care and exclusively breastfeeding.

To test our hypothesis that maternal participation in Chamas improved MNCH outcomes, including ECD, we conducted a cluster-randomized controlled trial in Trans Nzoia County, Kenya. In this article, we report results from developmental screening questionnaires (DSQ) completed on children born during the trial at 1-year follow-up.

## METHODS

### Study Design and Setting

We analyzed DSQ data from a 2-arm cluster randomized controlled trial in 74 communities across 4 subcounties (Cherangany, Saboti, Kwanza, and Kiminini) in Trans Nzoia County, Kenya. We chose a cluster-randomized design to minimize contamination due to intervention exposure between neighboring villages. We defined clusters as community health units—geographically defined health service delivery areas for populations of 5,000 people overseen by community health volunteers (CHVs).^16^ We randomized community health units 1:1 (non-stratified, non-matched) using a simple random allocation sequence to participate in Chamas (intervention) or receive recommended monthly home visits from CHVs (standard of care) for 1 year. Data collectors, analysts, and investigators were masked to cluster allocation throughout the study; however, trial arms were identifiable to participants and CHVs by design.

We selected Trans Nzoia due to its geographic and socioeconomic diversity, as well as the presence of longstanding collaborations between the Government of Kenya, Ministry of Health, and Academic Model Providing Access to Healthcare. Trans Nzoia has nearly 1 million residents who largely subside on agricultural businesses and raising livestock. Moreover, health indicators for mothers and infants are consistently poorer than national estimates, reflecting a need for increased attention to MNCH policy and programming.^17^

### Procedures

Pregnant women presenting to the local health facility for their first antenatal care visit by 32 weeks' gestation were eligible to participate in the parent trial. Participants were allocated to each arm by their randomized community of residence. At approximately 1-year follow-up (i.e., 12 months of Chamas participation, initiating prenatally), we screened all children born during the trial with the DSQ, with no additional exclusion criteria. Data collectors traveled to participant homes to collect in-person data using electronic tablet-based, structured questionnaires. We synced data at the end of each collection day to a central, encrypted server. Research assistants made 3 attempts to contact participants over a 2-week period before declaring them lost to follow-up.

Intervention details are described in our protocol (ClinicalTrials.gov NCT03187873)^18^ and previous publications. A detailed summary of the intervention is noted within Supplement 1. Briefly, Chamas clusters convened twice per month for 12 months for group-based health lessons led by CHVs. Each group typically included 15–20 women, their infants, 2 CHV facilitators, and 2 postmenopausal mentor mothers. Health lessons during the first year of the program promoted positive MNCH behaviors during pregnancy (e.g., attending adequate antenatal care) and infancy (e.g., exclusively breastfeeding and immunizing infants). Women were also introduced to topics contributing to risk factors and social determinants associated with developmental delays such as infant growth monitoring and nutrition,^1^^,^^19^ disease prevention,^20^^,^^21^ childhood harsh punishment,^22^ and parental stress.^14^^,^^23^ These and other developmentally focused lessons are largely addressed during the second and third years of the curriculum. After each lesson, women were also invited to participate in an optional microfinance program called Group Integrated Savings for Health and Empowerment (GISHE). Participation in GISHE was completely optional so as not to deter women without financial means to contribute to group savings from joining Chamas. Women who chose to participate were encouraged to use savings and loans generated by GISHE to enroll in health insurance, pay for school fees and educational materials, and/or start small businesses.

Control clusters had monthly CHV home visits during the antenatal and postpartum period as recommended by the current standard of care.^25^ During monthly visits, CHVs aimed to collect basic health information, recognize antenatal and early postpartum danger signs, aid in infant growth monitoring, and refer individuals requiring services to health facilities. Further, CHVs were expected to encourage women to adopt the same positive health behaviors emphasized in Chamas, namely: attending antenatal care, delivering in health facilities, exclusively breastfeeding, adopting modern methods of contraception, fully immunizing infants, and ensuring adequate infant nutrition.

### Study Outcomes

Our primary outcome of interest was the rate of “at-risk development” across study arms. We defined “at-risk development” as any child who screened positive in 1 or more functional domains on the DSQ. We selected the DSQ as it has been validated for use among children less than aged 2 years in an LMIC.^26^ Of note, this tool is a screening questionnaire and not a diagnostic neurodevelopmental assessment. This validated questionnaire asks parents to report responses to 8 dichotomous “yes/no” questions in each of the following functional domains, which are specific to age (in months): gross motor, fine motor, vision, hearing, cognition, socialization, behavior, and speech. A list of the DSQ testing items can be viewed within Supplement 2. We considered any “yes” response a positive screen. Data collectors used age-appropriate DSQs for each infant by imposing an electronic checkpoint in RedCap (coded by birthdate). Lastly, if participants carried a multiple gestation pregnancy, we conducted independent questionnaires for each child.

Our primary outcome of interest was the rate of “at-risk development” across study arms.

To assess modifying effects of covariates, we collected baseline sociodemographic and reproductive health data for all participants. We used the validated Kenya 2015 Poverty Probability Index (PPI) tool to calculate individual poverty likelihood scores using the National Poverty Line Look-Up Table.^27^ This tool comprises 10 questions that assess sociodemographic factors such as county of residence, household education level, housing materials, and recent household purchases. Answers are coded using a numeric scoring system and summarized in a composite PPI score, which can be converted to a percentage value for poverty probability. We additionally collected end-line data on participant attitudes toward harsh punishment, infant birth weight, and age of first mixed-feeding as these variables have demonstrated significant associations with developmental delay outcomes in previous studies.^22^^,^^23^^,^^29^ We used a single item from the validated ISPCAN child abuse screening tool, parent version, to assess harsh punishment.^30^ Responses were collected using a 5-point Likert scale, ranging from “strongly disagree” to “strongly agree.”

### Statistical Analysis

The sample size calculation was based on the study's primary outcome, which was facility-based births.^14^ This calculation used methods described by Rutterford et al. for a proposed mixed-effects regression analysis^31^ using derived baseline estimates.^14^^,^^32^ We assumed a mean cluster size of 20 individuals, with 77 clusters (equally allocated between arms) and intracluster correlation coefficient of 0.44 (based on pilot data^14^), and 20% attrition. With these assumptions, a total of 1,280 individuals would be needed to detect a 4.7% difference on the risk difference scale with 80% power at a (2-tailed) significance level of .05. A total of 1,273 (66.3%) of participants completed the study at 12-month follow-up with DSQ data: we included 689 individuals from 37 clusters in the intervention (69.2%) and 584 individuals from 37 clusters in the control (63.2%) arms for analysis.

We assessed individual-level outcomes on all participants with complete DSQ data at 12-months follow-up. We used an intention-to-treat approach by evaluating all intervention participants, regardless of Chamas attendance. We summarized participant characteristics in a tabular form. We calculated frequencies and percentages for categorical variables, as well as means and standard deviations for continuous variables. We compared proportions using chi-squared tests and means using independent t-tests.

We compared proportions of at-risk development among children within each study arm using chi-square tests. We used simple descriptive statistics to determine DSQ testing items and domains for which children most commonly screened positive. We reviewed available sociodemographic as well as endline variables and identified potential confounders using clinical judgment and evidence from the literature. We performed a univariate analysis with each identified variable and included those demonstrating statistical significance (*P*<.05) or clinical meaning in our adjusted multivariate logistic regression model. We reported overall tests for each variable as well as estimated odds ratios with their 95% confidence intervals (CI). For households with twin children, we included DSQ data from the twin screened first in our regression model to limit bias introduced from duplicating covariate data from the same household and mother.

We decided a priori to restrict analyses solely to participants with both complete DSQ data and complete PPI data. We disaggregated items within the PPI to appropriately adjust for specific confounding variables known to impact child development (e.g., primary caregiver education level, head of household education level, housing materials).^32^^,^^33^ We conducted all statistical analyses in SAS 9.4 (SAS Institute, Cary, NC) statistical software and with α set to .05.

### Ethical Considerations

We prospectively registered the parent trial with ClinicalTrials.gov (NCT03187873). Our study received ethics approval from the Institutional Research Ethics Committee at Moi Teaching and Referral Hospital and Moi University (IREC/2018/269) and the Office of Research Administration at Indiana University (1905296355). We obtained written informed consent from participants before data collection.

### Role of the Funding Source

The funders had no role in the research design, collection, analysis, or interpretation of data, writing this report, or the decision to submit this manuscript for publication. The corresponding author had full access to all data in the study as well as final responsibility for the decision to submit this manuscript for publication.

## RESULTS

Between November 27, 2017, and March 8, 2018, we enrolled 1,920 pregnant women from 74 communities to participate in the parent trial. At 1-year follow-up, we screened 1,273 (689 intervention, 584 control) of their children with the DSQ (Figure). Among those without DSQ data, 36 children in the intervention arm and 42 children in the control arm died before follow-up. Lastly, all 12 twin pairs in our study cohort had concurrent DSQ screening results between the twins.

**FIGURE:**
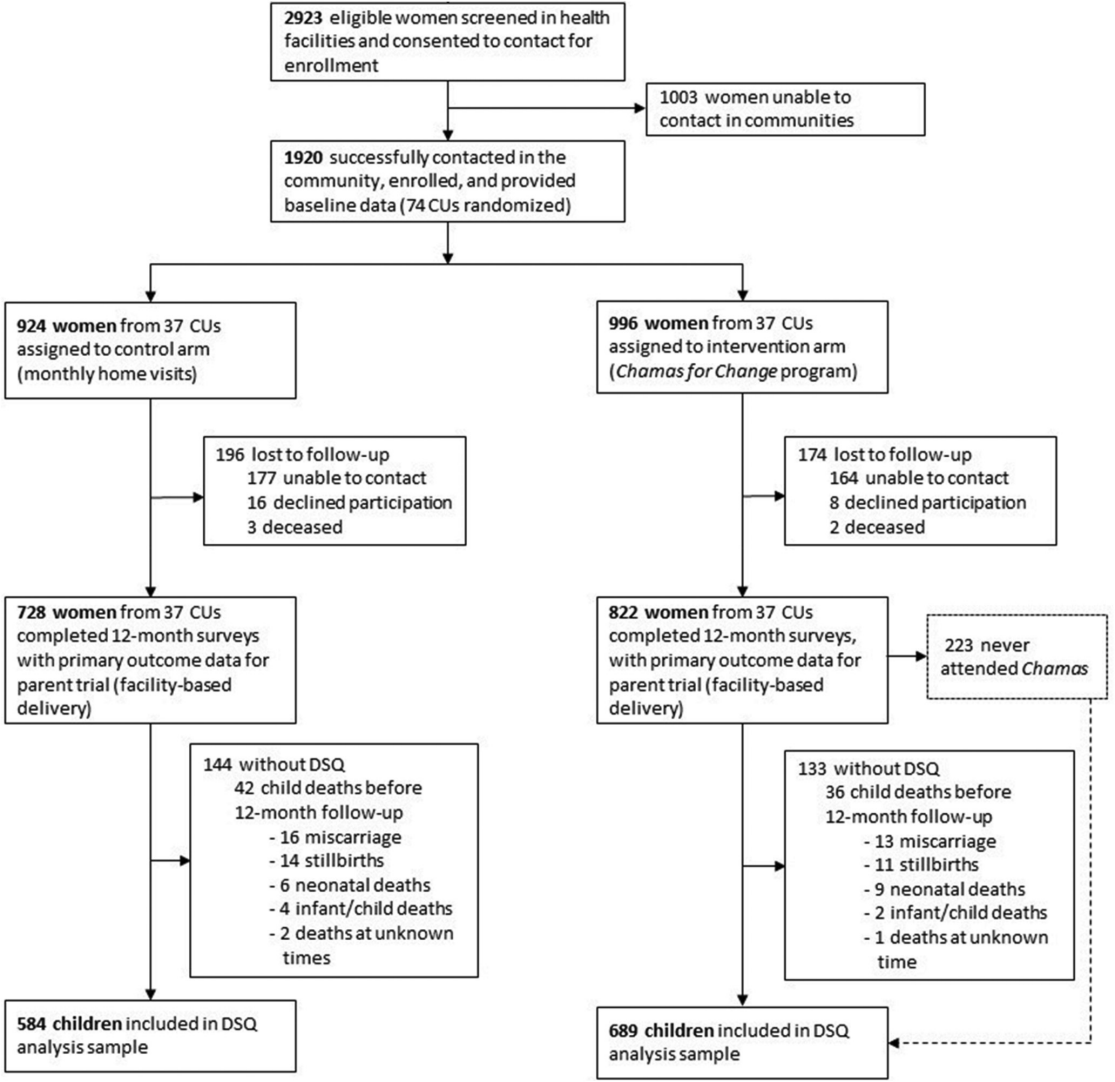
Cluster-Randomized Controlled Trial Profile and Study Inclusion Criteria of Maternal Participation in a Community-Based Women's Health Education Program in Rural Kenya Abbreviations: CUs, community health units; DSQ, developmental screening questionnaire; Chamas, Chamas for Change Program.

Table 1 summarizes sociodemographic data for our study population (N=1,273). Among included households, the mean child age was 10.9 months (standard deviation [SD]: 2.3) and maternal age was 26.8 years (SD: 6.4). The majority of mothers were married (83.8%, n=1,067) and unemployed (63.0%, n=802). Overall, arms were fairly balanced; however, controls had more household members educated beyond primary school (41.1% vs. 37.6%; *P*<.001) and a lower poverty likelihood (29.0% vs. 32.0%; *P*=.007) compared to intervention participants. Participants lost to follow-up were similar in number across study groups and attrition was not significantly associated with sociodemographic characteristics.

**TABLE 1.** Characteristics of Women Included in a Cluster-Randomized Study on the Effect of Maternal Participation in a Women's Health Education Intervention on Early Childhood Development, Tranz Nzoia County, Kenya

	Total (N=1,273) N (%)	Intervention (n=689) n (%)	Control (n=584) n (%)	P Value
Child's age, months, mean (SD)	10.9 (2.3)	11.2 (2.4)	10.5 (2.2)	<.001
Mother's age, years, mean (SD)	26.8 (6.4)	27.0 (6.6)	26.7 (6.1)	.341
Categorized mother's age, years				
<18	48 (3.8)	32 (4.8)	16 (2.8)	
18–24	462 (36.8)	240 (35.8)	222 (38.1)	
25–32	487 (38.8)	259 (38.6)	228 (39.2)	
>32	256 (20.4)	140 (20.9)	116 (19.9)	.266
Missing data (n)	20	18	2	
Marital status				
Single/divorced/separation/widowed	206 (16.2)	117 (17.0)	89 (15.2)	
Married	1067 (83.8)	572 (83.0)	495 (84.8)	.401
Employment status				
Unemployed	802 (63.0)	437 (63.4)	365 (62.5)	
Temporary work	74 (5.8)	41 (6.0)	33 (5.7)	
Self-employed/permanently employed	397 (31.2)	211 (30.6)	186 (31.9)	.885
Highest educational level of household head*				
Pre-primary/none/other	181 (14.3)	87 (12.7)	94 (16.1)	
Primary	592 (46.6)	342 (49.9)	250 (42.8)	
Secondary/post-primary/ vocational	391 (30.8)	218 (31.9)	173 (29.6)	
College or higher	106 (8.4)	39 (5.7)	67 (11.5)	
Missing data (n)	3	3	0	<.001
Highest educational level of any member^a^				
Pre-primary/none/other	103 (8.2)	44 (6.5)	59 (10.1)	
Primary	485 (38.5)	268 (39.6)	217 (37.2)	
Secondary/post-primary/vocational	468 (37.1)	272 (40.2)	196 (33.6)	
College or higher	205 (16.3)	93 (13.7)	112 (19.2)	.002
Missing data (n)	12	12	0	
Household food access (during last 7 days)^a^				
Bread	834 (65.8)	439 (64.3)	395 (67.6)	.209
Missing data (n)	6	6	0	
Meat/fish	924 (72.8)	488 (71.2)	436 (74.7)	.173
Missing data (n)	4	4	0	
Bananas	782 (62.0)	405 (59.6)	377 (64.8)	.057
Missing data (n)	11	9	2	
Household items^a^				
Towels	761 (59.9)	398 (58.0)	363 (62.2)	.134
Missing data (n)	3	3	0	
Thermos flask	899 (71.1)	492 (72.1)	407 (69.8)	.362
Missing data (n)	8	7	1	
Housing: wall material^a^				
Natural walls	994 (78.2)	561 (81.7)	433 (74.1)	
Finished walls	262 (20.6)	124 (18.1)	138 (23.6)	
Uncovered wall	15 (1.2)	2 (0.3)	13 (2.2)	<.001
Missing data (n)	2	2	0	
Housing: floor material^a^				
Natural floor	933 (73.6)	523 (76.4)	4110 (70.3)	
Other	335 (26.4)	162 (23.7)	173 (29.7)	.015
Missing data (n)	5	4	1	
% poverty likelihood^b^ (at national poverty line^a^, mean (SD)	0.3 (0.2)	0.32 (0.20)	0.29 (0.21)	.007
Missing data (n)	134	94	49	

Abbreviation: SD, standard deviation.

a Indicates variables included within the poverty probability index.

b The % poverty likelihood was derived based on the participants who had non-missing values on variables with ^a^ in the table. We excluded 6 intervention and 6 control second-born twins among live dyads assessed.

The overall rate of at-risk development among our study cohort was 3.5% (n=45). When divided into study groups, we observed a significantly lower rate of at-risk development in the intervention arm (2.5% vs. 4.8%, *P*=.025). We noted variable rates of at-risk development across child age (in months) at the time of screening (Table 2). The only significantly different rate between study arms, however, occurred at 8 months of age (10% (4/40) control vs. 0% (0/46) intervention, *P*=.043). The highest proportion of positively screened items pertained to the speech (33.7%, 30/89); gross motor (18.0%, 16/89); and cognition (11.2%, 10/89) domains. All remaining domains—fine motor, vision, hearing, socialization, and behavior—individually comprised no more than 4.5%–10.1% of remaining positive screening items.

**TABLE 2.** Rates of Child At-Risk Development, by Age and Study Group, in a Cluster-Randomized Study on the Effect of Maternal Participation in a Women's Health Education Intervention on Early Childhood Development, Tranz Nzoia County, Kenya

Child Age (Months) (N=1,273)	Intervention	Control	P Value
1 (N=5)	0/2	2/3 (66.7%)	.400
2 (N=5)	0/2	0/3	--
3 (N=8)	0/5	1/3 (33.3%)	.375
4 (N=6)	1/3 (33.3%)	1/3 (33.3%)	1.000
5 (N=9)	0/6	0/3	--
6 (N=6)	2/2 (100.0%)	3/4 (75.0%)	1.000
7 (N=55)	2/29 (6.9%)	3/26 (11.5%)	.659
8 (N=86)	0/46	4/40 (10.0%)	.043
9 (N=132)	2/59 (3.4%)	4/73 (5.5%)	.691
10 (N=169)	0/78	4/91 (4.4%)	.125
11 (N=243)	1/114 (0.9%)	1/129 (0.8%)	1.000
12 (N=236)	3/114 (2.6%)	3/122 (2.5%)	1.000
13 (N=169)	2/120 (1.7%)	2/49 (4.1%)	.580
14 (N=102)	1/73 (1.4%)	0/29 (0)	1.000
15 (N=42)	3/36 (8.3%)	0/6	1.000
**TOTAL**	17/689 (2.5%)	28/584 (4.8%)	0.025

We observed a significantly lower rate of at-risk development in the intervention arm (2.5%) compared to the control arm (4.8%).

We recorded outcomes on maternal attitudes toward harsh punishment, age of first mixed-feeding, and infant birthweight to adjust for their modifying effects on at-risk development. Mothers in the intervention arm more commonly agreed or strongly agreed with the use of physical punishment to discipline their children (80.6% vs. 71.1%; *P*<.001). Mean infant birthweight differed slightly between study arms, however, both were clinically normal (3.5 kg control vs. 3.4 kg intervention, *P*=.036). Lastly, mothers in the intervention arm more commonly delayed mixed feedings after 3 months of age compared to controls (80.1% vs. 75.4%; n=1260; *P*=.049).

Multivariate logistic regression models demonstrated randomized study group (*P*=.03), housing wall material (*P*=.018), and child's age (in months) at screening (*P*<.001) were significantly associated with likelihood of at-risk development (Table 3). Specifically, infants in Chamas had lower odds of at-risk development compared to controls (OR=0.50; 95% CI=0.27, 0.94) (Table 4). Participants with natural or uncovered walls demonstrated lower odds of at-risk development than those with finished walls (OR=0.27; 95% CI=0.09, 0.80). Older infant age (in months) at the time of screening was also associated with a protective effect (OR=0.82; 95% CI=0.73, 0.92).

**TABLE 3.** Factors Associated With Child At-Risk Development in a Cluster-Randomized Study on the Effect of Maternal Participation in a Women's Health Education Intervention on Early Childhood Development, Tranz Nzoia County, Kenya

Factor	Degrees of Freedom	Wald Chi-square Test Statistic	P Value
Study randomization (intervention vs. control)	1	4.72	.030
Categorized mother's age in years	3	0.98	.807
Marital status	1	0.11	.739
Employment status	2	4.51	.105
Highest educational level of household head	3	4.81	.186
Housing-wall material	1	5.56	.018
Housing-floor material	1	3.74	.053
Age of mixed feeding	1	1.96	.162
Attitudes towards harsh punishment	2	1.65	.437
Child's birth weight in kilograms	1	3.54	.060
Child's age in months	1	12.52	<.001

**TABLE 4.** Factors Associated With Child At-Risk Development in a Cluster-Randomized Study on the Effect of Maternal Participation in a Women's Health Education Intervention on Early Childhood Development, Tranz Nzoia County, Kenya

	Odds Ratio	95% Confidence Interval
Study randomization		
Intervention^a^	0.50^b^	0.27, 0.94 ^b^
Control (reference)	-----	-------
Categorized mother's age (in years)		
<18	0.91	0.14, 5.84
18–24	1.42	0.57, 3.53
25–32	1.47	0.60, 3.62
>32 (reference)	------	-----
Marital status		
Single/divorced/separated/widowed	0.86	0.36, 2.05
Married (reference)	-----	-----
Employment status		
Unemployed	2.16	0.93, 5.00
Temporary work	3.30	0.97, 11.26
Self-employed/permanently employed (reference)	---	----
Highest educational level of household head		
Pre-primary/none	1.55	0.39, 6.11
Primary	1.60	0.47, 5.46
Secondary	0.65	0.18, 2.41
College or higher (reference)	-----	-------
Housing- wall material		
Natural/uncovered walls	0.27^b^	0.09, 0.80^b^
Finished walls (reference)	-----	-------
Housing- floor material		
Natural floor	3.02	0.97, 9.26
Other (reference)	-----	-------
Age of mixed feeding		
>3 months	0.63	0.33, 1.20
≤ 3 months (reference)	-----	-------
Attitudes towards harsh punishment (response to “To properly raise a child, one must use harsh punishment”)		
Strongly agree/ agree	5.71	0.40, 81.53
Disagree/strongly disagree	5.42	0.36, 82.04
Neither agree or disagree (reference)	-----	-----
Child's birth weight (in kilograms)	0.64	0.40, 1.02
Child's age in months (in months)^c^	0.82^b^	0.73, 0.92^b^

a Excluded 168 subjects due to missing values for primary outcome or covariate data, with 1,105 children remaining for analysis.

b Statistically significant with α set at .05.

c At the time of developmental screening questionnaire evaluation.

## DISCUSSION

In 2015, the inclusion of ECD in the United Nation's Sustainable Development Goals (SDGs) was a landmark in the history of global policy surrounding this issue. Protecting, supporting, and promoting ECD is essential to accomplishing the first SDG, “to ensure that all human beings can fulfill their potential in dignity and equality.”^34^ Despite invigorated efforts and national commitments to support ECD, however, programs globally remain challenged by multi-factorial obstacles including inadequate and uncertain funding, inefficient resource allocation, and persistent health disparities.^35^ Moreover, recent investments in sub-Saharan Africa have largely focused on bolstering early education for children aged 4–5 years; while important, these strategies miss a critical window to intervene during the first 1,000 days of life.^36^^,^^37^ In this context, we rigorously evaluated the effect of a community-based women's health education program on at-risk development among children in rural Kenya. Our intention-to-treat analysis using a large sample from a geographically diverse catchment demonstrated a significant protective association between Chamas participation and at-risk development. Specifically, infants in Chamas demonstrated half the odds of at-risk development compared to those whose mothers received Ministry of Health recommended home visits.

The Chamas model embraces a multipronged approach to enhancing MNCH outcomes, including ECD. By providing women with opportunities to gain health literacy in a peer supportive environment, Chamas empowers women with both knowledge and community to improve outcomes for themselves and their infants during a critical developmental period. Further, among a subset of women that also engaged in microfinance activities through GISHE, this approach may have also helped mitigate important social determinants of health (e.g., inadequate funds to pay for health services). Though the latter is not a focus of this report (and will be explored in future publications), we speculate all 3 components of the model significantly contribute to its success. Our findings offer evidence to support that by addressing health, providing community, and offering a means to combat social determinants, this intervention may help protect children from developmental delays in this resource-limited setting.

Chamas empowers women with both knowledge and community to improve outcomes for themselves and their infants during a critical developmental period.

It is important to note that at the point of DSQ testing, Chamas participants received limited education related to child development-focused topics, and instead, prioritized topics related to basic infant care, including proper nutrition, breastfeeding, watching for worrisome signs in the first 2 weeks of life, and the importance of setting routines, with 1 session on infant development. Interpreted in context, these findings underscore that when other factors known to impact child development are addressed, such as antenatal care attendance, facility-based delivery, exclusive breastfeeding, and adequate nutrition, rates of developmental delay may simultaneously decrease.^16^^,^^24^^,^^38^ This hypothesis is supported by literature from other LMICs that highlight a synergistic relationship between reinforcing positive MNCH behaviors and mitigating developmental delay risk.^28^^,^^39^ However, recent studies measured the effectiveness of 1 or 2 focused interventions on improving ECD outcomes and most have had mixed results. A recent trial that evaluated a water, sanitation, and hygiene intervention combined with nutritional counseling and supplementation in Zimbabwe demonstrated differential ECD improvement based on HIV status, with HIV-exposed children experiencing greater benefit than those uninfected.^40^^,^^41^ A home-based program in Pakistan that combined nutritional services and responsive caregiving training also demonstrated improvement in ECD outcomes.^42^^,^^43^ Very little data exist, however, on programs utilizing multiple concurrent strategies like in Chamas. As such, our findings build upon a growing body of evidence that supports MNCH strategies for mitigating developmental delay risk while also underscoring the importance of addressing its social determinants.

The rate of at-risk development was lower among infants in our cohort compared to population-based modeling estimates. Less than 4% of infants in our cohort demonstrated at-risk development, which is significantly lower than an estimated 45% in Kenya and 43% across LMICs.^3^^,^^5^ Of note, the national and international estimates of at-risk development are modeled using population data for extreme poverty and stunting as proxies for at-risk development, rather than individual-level evaluations, which generally yield greater precision. Even with more informed modeling, using data from health surveys, cohort studies, and hospital databases, the estimates of identified developmental disability in young Kenyan children is 10%,^44^ which is still higher than this trial's estimate. Due to the varying definitions of at-risk development or developmental disability and the data used for modeling, it is difficult to compare those values with individual-level screening with the DSQ. When our findings are compared to other studies using the DSQ, rates of at-risk development within this study are comparable, albeit, still slightly lower than expected. These studies determined rates of at-risk development of 4.8%–7.3% in children under age 2 years using the DSQ.^45^^,^^46^ Within the DSQ validation trial in Bangladesh, 17% of children tested were identified as at-risk for developmental delays.^26^ Because the DSQ was administered among a myriad of other questions, the risk for response bias and interpretability of questions across different cultural contexts may complicate these results. Additional investigations, such as formative work and cognitive interviews to ensure face validity of questions, testing alongside clinical examinations, or more detailed developmental assessments, are needed to evaluate the DSQ's adequacy in identifying infants with at-risk development in this setting.

Our study found that the majority of mothers in both the intervention and control groups agreed with using physical punishment to discipline their children, although this was more common among those in the intervention group. Corporal punishment as a discipline method is associated with worse child behavioral and developmental outcomes; however, most evidence is focused on outcomes of children older than those within our cohort (i.e., aged older than 5 years).^47^^–^^49^ Positive parenting training is integrated within the Chamas curriculum after 2 years of program enrollment, thus study participants had minimal exposure at the time of assessment. Mothers would have relied on their prior knowledge and experiences to guide their views on physical punishment, which is very common in Kenya,^50^ and the higher rates of physical punishment use among the intervention group was likely incidental. The long-term impact of childhood physical punishment and parental exposure to positive parenting on ECD should be explored within this cohort in future studies.

The majority of mothers in both the intervention and control groups agreed with using physical punishment to discipline their children, which is associated with worse child behavioral and developmental outcomes.

Aligned with current evidence, this study found that delayed introduction of mixed feeding was protective against at-risk development. Exclusive breastfeeding in the first 3–6 months of life has been associated with improved cognitive outcomes early in life; however, that benefit may not be maintained as children become school-aged.^51^^–^^53^ While the mechanism for this effect is unclear and likely multifactorial,^54^ delayed mixed feeding improves nutritional status.^55^ Malnutrition is a critical risk factor for worsened developmental outcomes.^23^ Although our analyses were limited by the absence of objective nutritional markers, we predict this adjustment would have minimally impacted our primary outcome. Children within the control group had a lower probability of poverty and, by proxy, would have been more likely to access nutritious foods. Future studies involving this cohort will work toward obtaining anthropometric measurements on study participants.

Moreover, our findings demonstrated that as the age of the child increased, the rates of at-risk development decreased. One potential explanation for this finding is related to the left-shifted or skewed distribution of age within this study cohort toward the upper end of the age range, allowing for a more reliable interpretation of the data through the model at older ages. Another potential explanation for this age-related difference is the enhanced reliability of testing in older children. The American Academy of Pediatrics recommends beginning developmental screening at 9 months, as many skills emerge by and differentiate at this age, resulting in a more reliable evaluation of developmental status.^56^ While less reliable, screening for at-risk development within younger aged children can be helpful to allow for those identified as having delays to be referred to appropriate therapies during the first 1,000 days of life, when they are most likely to gain the most benefit from the services.^36^ Longitudinal assessment of this cohort should be explored to understand the stability of developmental status over time.

An unexpected finding of our analysis was the decreased odds of at-risk development associated with uncovered or natural walls compared to finished walls. Finished walls are typically correlated with higher socioeconomic status, which has demonstrated association with improved developmental outcomes.^33^^,^^57^ There is evidence to suggest the quality of a child's home environment, such as the housing quality, residential mobility, and availability of learning materials, may impact ECD.^57^^,^^58^ However, few studies have looked at specific housing materials,^33^ and none have specified an association between wall material and ECD.^33^ Attempting to quantify poverty status in LMICs poses significant challenges and limitations. However, tools that incorporate multiple variables to differentiate individuals on economic statuses, such as the PPI, can be helpful. While it is unclear at this time why a finding associated with higher socioeconomic status is linked with increased odds of at-risk development, we hypothesize that more complex mediators may come into play when a family has finished walls. For example, families with finished walls within their home may have 2 income-earning parents, requiring childcare for the infants. In settings like Kenya, there are limited options for high-quality, stimulating childcare, a variable for which no data were collected for this study. Another potential mediating factor may be potential exposure to lead paint, which is still used in parts of Kenya^59^ and is known to be associated with worse developmental outcomes.^60^ However, these ideas are speculative in nature and should be further explored. More delineated knowledge about household factors and their implications on child development is needed.

An unexpected finding of our analysis was the decreased odds of at-risk development associated with uncovered or natural walls compared to finished walls.

Our study also has notable strengths. First, by using a cluster-randomized design, we strengthen the reliability as well as contextual relevance of our results for future implementation in a community-based setting. Second, we successfully recruited and collected data on a large cohort of participants across 4 diverse sub-counties in Trans Nzoia, strengthening the generalizability of our findings. Third, by conducting an intention-to-treat analysis, our findings suggest any degree of exposure to the Chamas intervention may yield protective benefits against developmental delay. This analysis approach helps simulate a real-world context where perfect program attendance is unexpected. Fourth, this cohort was sufficiently large to generate significant results in our outcome of interest. These results help strengthen our understanding of developmental delay and potentially associated risk factors in a resource-limited setting. Lastly, we used a validated questionnaire to assess ECD, which also expands upon limited data to support the utility of this screening tool in other LMICs.

### Limitations

Our study has several limitations. We experienced moderate loss to follow-up rates, highlighting challenges in retention and data collection. Women living within rural areas of Kenya tend to be more difficult to trace for follow-up due to limited details regarding the location of their residence (i.e., no street addresses) and lack of cell phone access or cellular network coverage. Individuals who are self-employed as casual laborers must frequently move for employment opportunities. We believe these are the major reasons for lost-to-follow-up within our cohort, but more formal analysis may be required. Another limitation to this study is that the DSQ requires mothers to subjectively affirm responses to a set of observed behaviors, which risks introduction of both response and recall biases. Screening mechanisms that triangulate data from both subjectively recorded data and observed behaviors would improve the reliability of these outcomes; however, it is worth noting that these evaluations are often cost-prohibitive, particularly in resource-limited settings. Lastly, some variables that may influence child development, such as anthropometrics and nutritional status, were not included within our follow-up evaluations due to limited trial resources. We hope future studies of this intervention will include these data to strengthen our analyses.

## POLICY IMPLICATIONS AND CONCLUSIONS

A significant advantage of the Chamas model is that it leverages existing resources (e.g., CHVs) and emphasizes collaborative investment from key stakeholders to ensure the program iteratively responds to the community's needs. While other group-based caregiver interventions have demonstrated positive results for ECD, many of these strategies introduce resource-related challenges that may compromise long-term quality and sustainability in these settings.^61^^,^^62^ A cost-effectiveness analysis of the Chamas model was performed and found to be US$46 per beneficiary, with both mother and infants included as beneficiaries, and would decrease to US$33 per beneficiary if continued within the same region over time (full analysis detailed in Supplement 3). Further, this analysis of DSQ data from a cluster randomized controlled trial demonstrated Chamas' potential to improve ECD outcomes. Our findings suggest this community-based intervention focused on health education for pregnant and postpartum women may support ECD as compared to the standard of care. As such, policy makers should consider the Chamas model when looking to revise current strategies to help protect children from developmental delay in this setting.

A significant advantage of the Chamas model is that it leverages existing resources and emphasizes collaborative investment from key stakeholders to ensure the program iteratively responds to the community's needs.

In summary, maternal participation in a community-based women's health education program was associated with lower rates of at-risk development among their children compared to the standard of care. Overall, rates of at-risk development were lower than expected for this population, warranting further investigation. Chamas may help protect children from developmental delay in rural Kenya and other resource-limited settings. Future studies are needed to clarify this association and to improve our model so that we may continue to support women and children across Kenya and in other resource-limited settings.

## Supplementary Material

20-00349-McHenry-Supplement-2.pdf

20-00349-McHenry-Supplement-1.pdf

20-00349-McHenry-Supplement-3.pdf

## Notes

**Peer Reviewed**

**First published online:** December 3, 2021.
