# Supplementary material for: Participation in a Community-Based Women's Health Education Program and At-Risk Child Development in Rural Kenya: Developmental Screening Questionnaire Results Analysis
Source: Glob Health Sci Pract. 2021 Dec 31;9(4):818–31. doi: 10.9745/GHSP-D-20-00349 (PMC8691876; doi:10.9745/GHSP-D-20-00349)
Supplement: 20-00349-McHenry-Supplement-2.pdf [file 20-00349-McHenry-Supplement-2.pdf]

Supplement to: McHenry MS, Maldonado LY, Yang Z, et al. Participation in a community-based women's health education program and at-risk child development in rural Kenya: developmental screening questionnaire results analysis. *Glob Health Sci Pract*. 2021;9(4). https://doi.org/10.9745/GHSP-D-20-00349

Development Screening Questionnaire (DSQ)

Age	Domain	Questions	Item No.
0 month	Gross motor	Can move head in supine or prone position	DSQ-1
	Fine motor	Opens hand intermittently	DSQ-2
	Vision	Reacts to light	DSQ-3
	Hearing	Can hear (i.e., startles, eyes open wide, blinks, changes facial expression to sound, turns eyes towards sound)	DSQ-4
	Cognition	Alerts to voice, touch, face	DSQ-5
	Socialization	Can feel attachment and affection (e.g., stops crying to listen to mother's voice, seeing mother's face or when in mother's lap)	DSQ-6
	Behavior	No excessive crying or irritability and sleeps well	DSQ-7
	Speech	Produces crying sounds	DSQ-8
1 month	Gross motor	Can move head in supine or prone position	DSQ_9
	Fine motor	Opens hand intermittently	DSQ-10
	Vision	Watches face	DSQ-11
	Hearing	Can hear (i.e., startles, eyes open wide, blinks, changes facial expression to sound, turns eyes towards sound)	DSQ-12
	Cognition	Regards familiar face when being fed/stops crying when picked up or spoken to	DSQ-13
	Socialization	Can feel attachment and affection (e.g., stops crying to listen to mother's voice, seeing mother's face or when in mother's lap)	DSQ-14
	Behavior	No excessive crying or irritability and sleeps well	DSQ-15
	Speech	Produces crying sounds	DSQ-16
2 months	Gross motor	Sitting- lifts head up occasionally	DSQ-17.
	Fine motor	Opens hand intermittently	DSQ-18
	Vision	Can see large sized objects, i.e., about 12 cm diameter	DSQ-19
	Hearing	Can hear (i.e., startles, eyes open wide, blinks, changes facial expression to sound, turns eyes towards sound)	DSQ-20
	Cognition	Has eye to eye contact/eyes follows moving toy	DSQ-21
	Socialization	Can feel attachment and affection (e.g., stops crying to listen to mother's voice, seeing mother's face or when in mother's lap)	DSQ-22
	Behavior	No excessive crying or irritability and sleeps well	DSQ-23
	Speech	Vocalizes sounds other than crying (e.g., throaty sounds like cooing, gurgling)	DSQ-24
3 months	Gross motor	Sitting – holds head erect for a considerable time	DSQ_25

Age	Domain	Questions	Item No.
	Fine motor	Hands open most of the time	DSQ-26
	Vision	Can see objects, i.e., a cup	DSQ-27
	Hearing	Can hear (i.e., startles, eyes open wide, blinks, changes facial expression, or turns eyes towards sound)	DSQ-28
	Cognition	Smiles when spoken to/vocalizes when spoken to	DSQ-29
	Socialization	Can feel attachment and affection (e.g., stops crying to listen to mother's voice, seeing mother's face or when in mother's lap)	DSQ-30
	Behavior	No excessive crying or irritability, and sleeps well	DSQ-31
	Speech	Vocalizes sounds other than crying (e.g., throaty sounds like cooing, gurgling)	DSQ-32
4 months	Gross motor	Sitting- holds head erect for a considerable time	DSQ-33
	Fine motor	Hands open and holds object in hand for several seconds	DSQ-34
	Vision	Can see objects, i.e., a cup	DSQ-35
	Hearing	Can hear (i.e., startles, eyes open wide, blinks, changes facial expression, or turns towards sound)	DSQ-36
	Cognition	Looks at object he/she is holding	DSQ-37
	Socialization	Can feel attachment and affection (e.g., stops crying to listen to mother's voice, seeing mother's face or when in mother's lap)	DSQ-38
	Behavior	No excessive crying or irritability, and sleeps well	DSQ-39
	Speech	Vocalizes vowel sounds (e.g., aa, ee, oo)	DSQ-40
5 months	Gross motor	Full neck control present	DSQ-41
	Fine motor	Can reach and grasp object in front	DSQ-42
	Vision	Can see objects, i.e., a cup	DSQ-43
	Hearing	Can hear (e.g., startles, eyes open wide, blinks, changes facial expression, or turns towards sound)	DSQ-44
	Cognition	Reaches and grasps toy in front and shakes unintentionally	DSQ-45
	Socialization	Can feel attachment and affection (e.g., stops crying to listen to mother's voice, seeing mother's face or when in mother's lap)	DSQ-46
	Behavior	No excessive crying or irritability and sleeps well	DSQ-47
	Speech	Vocalizes vowel sounds (e.g., aa, ee, oo)	DSQ-48
6 months	Gross motor	Full neck control present	DSQ-49
	Fine motor	Can reach and grasp object, i.e., 1 inch size, by palmar grasp	DSQ_50
	Vision	Can see small objects, e.g., a smartie	DSQ-51
	Hearing	Can hear (i.e., startles, eyes open wide, blinks, changes facial expression, or turns towards sound)	DSQ-52

Age	Domain	Questions	Item No.
	Cognition	Knows familiar people (mum, dad)	DSQ-53
	Socialization	Can feel attachment and affection (e.g., stops crying to listen to mother's voice, seeing mother's face or when in mother's lap)	DSQ-54
	Behavior	Responsive to surroundings and sleeps well	DSQ-55
	Speech	Vocalizes syllables singly (e.g., ma, da, ba)	DSQ-56
7 months	Gross motor	Full neck control present and can go from supine to prone	DSQ-57
	Fine motor	Can reach and grasp object, i.e., 1 inch size, by palmar grasp and can transfer object from one hand to another	DSQ-58
	Vision	Can see small objects, e g. a smartie	DSQ-59
	Hearing	Can hear (e.g., startles, eyes open wide, blinks, changes facial expression, or turns towards sound)	DSQ-60
	Cognition	Knows familiar people (mum, dad)	DSQ-61
	Socialization	Can feel attachment and affection (e.g., stops crying to listen to mother's voice, seeing mother's face or when in mother's lap)	DSQ-62
	Behavior	Responsive to surroundings and sleeps well	DSQ-63
	Speech	Vocalizes syllables singly (e.g., ma, da, ba)	DSQ-64
8 months	Gross motor	Full neck control present and can go from supine to prone and can sit alone momentarily (2min)	DSQ-65
	Fine motor	Can reach and grasp object, i.e., 1 inch size, by palmar grasp and can transfer object from one hand to another	DSQ-66
	Vision	Can see small objects, e.g., a smartie	DSQ-67
	Hearing	Can hear (e.g., startles, eyes open wide, blinks, changes facial expression, or turns towards sound)	DSQ-68
	Cognition	Knows familiar people (mum, dad)	DSQ-69
	Socialization	Can feel attachment and affection (e.g., stops crying to listen to mother's voice, seeing mother's face or when in mother's lap)	DSQ-70
	Behavior	Responsive to surroundings and sleeps well	DSQ-71
	Speech	Vocalizes syllables singly (e.g., ma, da, ba)	DSQ-72
9 months	Gross motor	Full neck control present and can sit alone for few minutes (5-10min)	DSQ-73
	Fine motor	Can grasp object, e g. biscuit, puffed rice with fingers	DSQ-74
	Vision	Can see small objects, e.g., a smartie	DSQ-75
	Hearing	Can hear (i.e turns towards sound, or responds to call from out of sight)	DSQ-76
	Cognition	Beginning to distinguish strangers from familiar people	DSQ-77

Supplement to: McHenry MS, Maldonado LY, Yang Z, et al. Participation in a community-based women's health education program and at-risk child development in rural Kenya: developmental screening questionnaire results analysis. *Glob Health Sci Pract*. 2021;9(4). https://doi.org/10.9745/GHSP-D-20-00349

Age	Domain	Questions	Item No.
	Socialization	Can feel attachment and affection (e.g., stops crying to listen to mother's voice, seeing mother's face or when in mother's lap)	DSQ-78
	Behavior	Responsive to surroundings and sleeps well	DSQ-79
	Speech	Vocalizes double syllables (e.g., ma ma, da da, ba ba)	DSQ-80
10 months	Gross motor	Can sit alone steadily without risk of falling	DSQ-81
	Fine motor	Can grasp object, e.g., biscuit, puffed rice with fingers	DSQ-82
	Vision	Can see small objects, e.g., a smartie	DSQ-83
	Hearing	Can hear (i.e..turns towards sound or responds to call from out of sight)	DSQ-84
	Cognition	Clearly distinguishes strangers from familiar people	DSQ-85
	Socialization	Can feel attachment and affection (e.g., stops crying to listen to mother's voice, seeing mother's face or when in mother's lap)	DSQ-86
	Behavior	Responsive to surroundings and sleeps well	DSQ-87
	Speech	Babbles in strings (e.g., ma ma ma ma , da da da da)	DSQ-88
11 months	Gross motor	Can sit from lying and can crawl or bottom shuffle to move forward	DSQ-89
	Fine motor	Can grasp object, e.g., biscuit, puffed rice with fingers	DSQ-90
	Vision	Can see small objects, e.g., a smartie	DSQ-91
	Hearing	Can hear (i.e..turns towards sound, or responds to call from out of sight)	DSQ-92
	Cognition	Claps hands or wave bye-bye in imitation of adult/rings rattle purposefully	DSQ-93
	Socialization	Can feel attachment and affection (e.g., stops crying to listen to mother's voice, seeing mother's face or when in mother's lap)	DSQ-94
	Behavior	Responsive to surroundings and sleeps well	DSQ-95
	Speech	Babbles in strings (e.g., ma ma ma ma , da da da da)	DSQ-96
12 months	Gross motor	Can stand , holding on, from sitting position and can cruise around furniture	DSQ-97
	Fine motor	Can grasp object, e.g., biscuit, puffed rice with two fingers	DSQ-98
	Vision and	Can see small objects, e.g., a mustard seed/a lentil/ an ant	DSQ-99
	Hearing	Can hear (i.e.. turns towards sound, or responds to call from out of sight)	DSQ-100
	Cognition	Demonstrates affection on request /Plays pat-a-cake or waves bye-bye on request	DSQ-101
	Socialization	Responds when spoken to	DSQ-102
	Behavior	Responsive to surroundings and sleeps well	DSQ-103
	Speech	Vocalizes imitative or meaningless words (e.g., mom, dad)	DSQ-104
13 months	Gross motor	Can stand , holding on, from sitting position and can cruise around furniture	DSQ-105
	Fine motor	Can grasp object, e.g., biscuit, puffed rice with two fingers	DSQ-106

Supplement to: McHenry MS, Maldonado LY, Yang Z, et al. Participation in a community-based women's health education program and at-risk child development in rural Kenya: developmental screening questionnaire results analysis. *Glob Health Sci Pract*. 2021;9(4). https://doi.org/10.9745/GHSP-D-20-00349

Age	Domain	Questions	Item No.
	Vision	Can see small objects, e.g., a mustard seed/a lentil/ an ant	DSQ-107
	Hearing	Can hear (i.e.turns towards sound, or responds to call from out of sight)	DSQ-108
	Cognition	Demonstrates affection on request /Plays pat-a-cake or wave bye-bye on request	DSQ-109
	Socialization	Responds when spoken to	DSQ-110
	Behavior	Responsive to surroundings and sleeps well	DSQ-111
	Speech	Vocalizes imitative or meaningless words (e.g., mom, dad)	DSQ-112
14 months	Gross motor	Can stand , hold on, from sitting position and can cruise around furniture	DSQ-113
	Fine motor	Can grasp object, e.g., biscuit, puffed rice with two fingers	DSQ-114
	Vision	Can see small objects, e.g., a mustard seed/a lentil/ an ant	DSQ_115
	Hearing	Can hear (i.e., turns towards sound, or responds to call from out of sight)	DSQ-116
	Cognition	Demonstrates affection on request /Plays pat-a-cake or waves bye-bye on request	DSQ-117
	Socialization	Responds when spoken to	DSQ-118
	Behavior	Responsive to surroundings and sleeps well	DSQ-119
	Speech	Vocalizes imitative or meaningless words (e.g., mom, dad)	DSQ-120
15 months	Gross motor	Can stand alone without support from sitting	DSQ-121
	Fine motor	Can grasp object, e.g., biscuit, puffed rice with two fingers	DSQ-122
	Vision	Can see small objects, e.g., a mustard seed/a lentil/ an ant	DSQ-123
	Hearing	Can hear (i.e., turns towards sound or responds to call from out of sight)	DSQ-124
	Cognition	Points to or looks at familiar persons when requested/Understands and obeys simple instructions	DSQ-125
	Socialization	Responds when spoken to	DSQ-126
	Behavior	Attentive to situation (e.g., eating, playing. greeting, goodbye)	DSQ-127
	Speech	Vocalizes at least one meaningful word	DSQ-128
16 months	Gross motor	Can stand alone without support from sitting	DSQ-129
	Fine motor	Can grasp object, e.g., biscuit, puffed rice with two fingers	DSQ-130
	Vision	Can see small objects, e.g., a mustard seed/a lentil/ an ant	DSQ_131
	Hearing	Can hear (i.e., turns towards sound, or responds to call from out of sight)	DSQ-132
	Cognition	Points to or looks at familiar persons when requested/Understands and obey simple instructions	DSQ-133
	Socialization	Responds when spoken to	DSQ-134
	Behavior	Attentive to situation (e.g., eating, playing, greeting, goodbye)	DSQ-135
	Speech	Vocalizes at least two meaningful words	DSQ-136
17 months	Gross motor	Can stand alone without support from sitting	DSQ-137

Supplement to: McHenry MS, Maldonado LY, Yang Z, et al. Participation in a community-based women's health education program and at-risk child development in rural Kenya: developmental screening questionnaire results analysis. *Glob Health Sci Pract*. 2021;9(4). https://doi.org/10.9745/GHSP-D-20-00349

Age	Domain	Questions	Item No.
	Fine motor	Can grasp object, e.g., biscuit, puffed rice with two fingers	DSQ-138
	Vision	Can see small objects, e.g., a mustard seed/a lentil/ an ant	DSQ-139
	Hearing	Can hear (i.e., turns towards sound or responds to call from out of sight)	DSQ-140
	Cognition	Points to or looks at familiar persons when requested/Understands and obey simple instructions	DSQ-141
	Socialization	Responds when spoken to	DSQ-142
	Behavior	Attentive to situation (e.g., eating, playing. greeting, goodbye)	DSQ-143
	Speech	Vocalizes at least two meaningful words	DSQ-144
18 months	Gross motor	Can stand alone without support from sitting and can walk few steps	DSQ-145
	Fine motor	Feeds self	DSQ-146
	Vision	Can see small objects, e.g., a mustard seed/a lentil/ an ant	DSQ-147
	Hearing	Can hear (i.e., turns towards sound or responds to call from out of sight)	DSQ-148
	Cognition	Points to at least one body part if asked/Recognizes and/or point to named object or picture	DSQ-149
	Socialization	Interest in others (e.g., enjoys peer play, involves others in two-way play, understands others emotions, shares enjoyment with others)	DSQ-150
	Behavior	Attentive to situation (e.g., eating, playing, greeting, goodbye)	DSQ-151
	Speech	Vocalizes four meaningful words	DSQ-152
19 months	Gross motor	Can stand alone without support from sitting and can walk few steps	DSQ-153
	Fine motor	Feeds self	DSQ-154
	Vision	Can see small objects, e.g., a mustard seed/a lentil/ an ant	DSQ-155
	Hearing	Can hear (i.e., turns towards sound or responds to call from out of sight)	DSQ-156
	Cognition	Recognizes and/or point to named object or picture	DSQ_157
	Socialization	Interest in others (e.g., enjoys peer play, involves others in two-way play, understands others emotions, shares enjoyment with others)	DSQ-158
	Behavior	Attentive to situation (e.g., eating, playing, greeting, goodbye)	DSQ-159
	Speech	Vocalizes four meaningful words	DSQ-160
20 months	Gross motor	Can stand alone without support from sitting and can walk few steps	DSQ-161
	Fine motor	Feeds self	DSQ-162
	Vision	Can see small objects, e.g., a mustard seed/a lentil/ an ant	DSQ-163
	Hearing	Can hear (i.e.. turns towards sound or responds to call from out of sight)	DSQ-164
	Cognition	Recognizes and/or point to named object or picture	DSQ-165
	Socialization	Interest in others (e.g., enjoys peer play, involves others in two-way play, understands others emotions, shares enjoyment with others)	DSQ-166

Supplement to: McHenry MS, Maldonado LY, Yang Z, et al. Participation in a community-based women's health education program and at-risk child development in rural Kenya: developmental screening questionnaire results analysis. *Glob Health Sci Pract*. 2021;9(4). https://doi.org/10.9745/GHSP-D-20-00349

Age	Domain	Questions	Item No.
	Behavior	Attentive to situation (e.g., eating, playing, greeting, goodbye)	DSQ-167
	Speech	Vocalizes four meaningful words	DSQ-168
21 months	Gross motor	Can stand alone without support from sitting and can walk few steps	DSQ-169
	Fine motor	Feeds self	DSQ-170
	Vision	Can see small objects, e.g., a mustard seed/a lentil/ an ant	DSQ-171
	Hearing	Can hear (i.e.. turns towards sound or responds to call from out of sight)	DSQ-172
	Cognition	Recognizes and/or point to named object or picture	DSQ-173
	Socialization	Interest in others (e.g., enjoys peer play, involves others in two-way play, understands others emotions, shares enjoyment with others)	DSQ-174
	Behavior	Attentive to situation (e.g., eating, playing, greeting, goodbye)	DSQ-175
	Speech	Vocalizes four meaningful words	DSQ-176
22 months	Gross motor	Can stand alone without support from sitting and can walk few steps	DSQ-177
	Fine motor	Feeds self	DSQ-178
	Vision	Can see small objects, e.g., a mustard seed/a lentil/ an ant	DSQ-179
	Hearing	Can hear (i.e., turns towards sound, or responds to call from out of sight)	DSQ-180
	Cognition	Recognizes and/or point to named object or picture	DSQ-181
	Socialization	Interest in others (e.g., enjoys peer play, involves others in two-way play, understands others emotions, shares enjoyment with others)	DSQ-182
	Behavior	Attentive to situation (e.g., eating, playing, greeting, goodbye)	DSQ-183
	Speech	Vocalizes four meaningful words	DSQ-184
23 months	Gross motor	Can stand alone without support from sitting and can walk few steps	DSQ-185
	Fine motor	Drinks from glass	DSQ-186
	Vision	Can see small objects, e.g., a mustard seed/a lentil/ an ant	DSQ-187
	Hearing	Can hear (i.e.. turns towards sound or responds to call from out of sight)	DSQ-188
	Cognition	Recognizes and/or points to named objects (e.g., cup, doll, toothbrush, comb) or picture	DSQ-189
	Socialization	Interest in others (e.g., enjoys peer play, involves others in two-way play, understands others emotions, shares enjoyment with others)	DSQ-190
	Behavior	Attentive to situation (e.g., eating, playing, greeting, goodbye)	DSQ-191
	Speech	Vocalizes four meaningful words	DSQ-192

Obtained from:

Supplement to: McHenry MS, Maldonado LY, Yang Z, et al. Participation in a community-based women's health education program and at-risk child development in rural Kenya: developmental screening questionnaire results analysis. *Glob Health Sci Pract*. 2021;9(4). https://doi.org/10.9745/GHSP-D-20-00349

Khan, N.Z., Muslima, H., Shilpi, A.B., Begum, D., Akhtar, S., Parveen, M., Ferdous, S., McConachie, H. and Darmstadt, G.L. (2013), Validation of a home-based neurodevelopmental screening tool for under 2-year-old children in Bangladesh. Child: Care, Health and Development, 39: 643-650. doi:10.1111/j.1365-2214.2012.01393.x
