# Supplementary material for: Participation in a Community-Based Women's Health Education Program and At-Risk Child Development in Rural Kenya: Developmental Screening Questionnaire Results Analysis
Source: Glob Health Sci Pract. 2021 Dec 31;9(4):818–31. doi: 10.9745/GHSP-D-20-00349 (PMC8691876; doi:10.9745/GHSP-D-20-00349)
Supplement: 20-00349-McHenry-Supplement-1.pdf [file 20-00349-McHenry-Supplement-1.pdf]

Supplement to: McHenry MS, Maldonado LY, Yang Z, et al. Participation in a community-based women's health education program and at-risk child development in rural Kenya: developmental screening questionnaire results analysis. *Glob Health Sci Pract*. 2021;9(4). https://doi.org/10.9745/GHSP-D-20-00349

Supplement 1. Description of the Intervention

"The intervention clusters of this study participated in the Chamas program. *Chamas* is a group-based, community health volunteer (CHV)-led health education program that supports women and their infant children within the first 1,000 days of their life. Women who are randomized to the intervention arm participated in *Chamas* instead of the standard of care, which consists of individual home-visits. Participants attend group sessions twice a month, most lasting 60-90 minutes/each. The discussions during these sessions were focused on topics relevant to antenatal, postpartum, and early childhood experiences. Intervention CHVs used an illustrated flip-chart with evidence-based, structured discussion guides to facilitate lessons. Groups were typically comprised of 15-20 women, two CHV facilitators, and two mentor mothers, the latter being post-menopausal, experienced mothers. The first year of the curriculum promoted behaviors associated with reduced maternal and infant morbidity and mortality (such as attending ANC visits, delivering in health facilities, and exclusively breastfeeding). These lessons purposefully mirror health topics CHVs are expected to promote during individual home-visits under the community health support (CHS), which was the standard of care that control clusters were expected to receive. Within the intervention clusters, women were also invited to participate in an optional table-banking program called *Group Integrated Savings for Health and Empowerment (GISHE)*. Emphasis was placed on the fact that *GISHE* participation was optional, to not deter participation in the *Chamas* groups for those who did not have the financial means to participate in *GISHE*. so as not to deter women without financial means to contribute to group savings from joining *Chamas*. Women were encouraged to use savings generated by *GISHE* to finance health interventions (e.g., enroll in health insurance, pay for transportation to health facilities), invest in early childhood education, and/or start small businesses, however there was no requirement or restrictions on use of the funds.

Strategies to ensure fidelity of *Chamas* included: using standardized intervention materials (i.e. printed curriculum flipcharts), hosting structured CHV training sessions preceding the trial, offering monthly supervision by study staff, and designating at least two trained CHVs to every group to avoid potential disruptions due to illnesses or job transfers. In addition to attending the four-day MNCH refresher training, CHVs facilitating *Chamas* also received a formal two-day orientation to the program and were trained in group facilitation techniques. CHV facilitators were provided schedule support session throughout the trial (during months 1-3, 6, 9, and 12), which provided opportunities for feedback and communal trouble-shooting to enhance program delivery.

Control clusters had monthly CHV home-visits during pregnancy and postpartum, as recommended by the Kenyan CHS standard of care.[1] During monthly visits, CHVs collect basic health information, identify antenatal and early postpartum danger signs, refer individuals to care (if indicated), and aid in infant growth monitoring. CHVs are also expected to encourage women to adopt the same key health behaviors promoted in *Chamas*. CHVs working within control clusters received oversight and supervision from Community Heath Extension Workers, as structured by the CHS. CHVs performing door-to-door visits typically oversee a catchment of 15 women who are seen individually for 20-30 minutes on a monthly basis (up to 7.5 hours of volunteer effort per month). Those facilitating *Chamas* transitioned to group sessions in substitution of individual door-to-door visits; thus, their volunteer effort was reduced to two 60-90 minute sessions per month (up to 3 hours of volunteer effort per month).

Supplement to: McHenry MS, Maldonado LY, Yang Z, et al. Participation in a community-based women's health education program and at-risk child development in rural Kenya: developmental screening questionnaire results analysis. *Glob Health Sci Pract*. 2021;9(4). https://doi.org/10.9745/GHSP-D-20-00349

We did not provide incentives (monetary or other) for participation to CHVs, Community Heath Extension Workers or participants in either study arm at any point during the trial. CHEWs continued to receive their salaries from the Ministry of Health and CHVs, who volunteered their time outside of their other jobs (i.e. as teachers, farmers, laborers) were not prohibited from continuing to work throughout the trial. Notably, CHVs under the current CHS are not financially compensated for performing door-to-door visits. To reduce potential for confounding, we similarly did not compensate CHVs for facilitating *Chamas* meetings. All CHVs and Community Heath Extension Workers were, however, provided with reimbursements for travel to meetings and trainings as well as cell phone minutes used to contact participants during trial recruitment."

1 Ministry of Health Division of Community Health Services. *Community Health Volunteers (CHVs): Basic Modules Handbook.* Nairobi, Kenya: USAID;2013.
