# Supplementary material for: Participation in a Community-Based Women's Health Education Program and At-Risk Child Development in Rural Kenya: Developmental Screening Questionnaire Results Analysis
Source: Glob Health Sci Pract. 2021 Dec 31;9(4):818–31. doi: 10.9745/GHSP-D-20-00349 (PMC8691876; doi:10.9745/GHSP-D-20-00349)
Supplement: 20-00349-McHenry-Supplement-3.pdf [file 20-00349-McHenry-Supplement-3.pdf]

Supplement to: McHenry MS, Maldonado LY, Yang Z, et al. Participation in a community-based women's health education program and at-risk child development in rural Kenya: developmental screening questionnaire results analysis. *Glob Health Sci Pract*. 2021;9(4). https://doi.org/10.9745/GHSP-D-20-00349

Supplement 3. A Proposed Cost-Effectiveness Analysis of the Chamas for Change Model in Western Kenya

Cost of Chamas Intervention

To identify and evaluate costs of the *Chamas* intervention, we followed guidance provided by USAID's Maternal and Child Survival Program's report, "Costs and cost-effectiveness of community health investments in reproductive, maternal, neonatal, and child health."[1] We categorized inputs and associated costs into "start-up" and "recurrent" inputs/costs. These inputs and costs were associated with delivering the intervention and not the inputs and costs associated with conducting the study. Study-specific costs such as hiring study research assistants for data collection were not included. Inputs and costs (provided in US dollars, USD) for the intervention are described below in Table 1.

Table 1. Inputs and Costs of the Chamas Intervention

Input	Cost Item(s)	Amount (US$)	Justification
**Startup costs**			
Stakeholder sensitization	Meetings with health officials (transportation, food, etc.)	4,257	The program met quarterly with County health officials to educate them on the *Chamas* intervention and obtain buy in. This was necessary because the intervention relies on County support for *Chamas* as part of their Community Health Strategy and to approve training and utilizing CHVs for delivering the intervention. Rather than their existing mandate to provide home-based visits to pregnant women under the Community Health Strategy, our program provides CHVs the opportunity to conduct group-based visits with women while they are facilitating *Chamas*.
	Meetings with community leaders (transportation, food, etc.)	777	Given that this intervention takes place in the community, our program also requires ensuring that community leaders and representatives such as Chiefs and Assistant Chiefs are supportive of the intervention.
Training of CHVs	Initial training for CHVs on *Chamas* curriculum (transportation, food, stipends)	3,516	This was a 3-day training for CHVs as well as Community Health Extension Workers (CHEW), who are Ministry of Health employees and provide supervision for CHVs. The focus on the training was on the *Chamas* curriculum and how to facilitate group-based education and care. A

[1] Costs and cost-effectiveness of community health investments in reproductive, maternal, neonatal, and child health. March 2017. USAID Maternal and Child Survival Program. Available online at: https://www.mcsprogram.org/resource/costs-cost-effectiveness-community-health-investments-reproductive-maternal-neonatal-child-health/

Supplement to: McHenry MS, Maldonado LY, Yang Z, et al. Participation in a community-based women's health education program and at-risk child development in rural Kenya: developmental screening questionnaire results analysis. *Glob Health Sci Pract*. 2021;9(4). https://doi.org/10.9745/GHSP-D-20-00349

			total of 74 CHVs and 125 CHEWs participated in the training.
Intervention supplies	Curriculum and Gishe boxes for each *Chamas* group	12,650	For facilitating *Chamas* groups, CHVs are provided a laminated flip chart that guides CHVs and participants through different modules (e.g., maternal health, immunizations, etc.). For the micro-finance table banking aspect of the group model, *Chamas* groups are also provided with a secure lockable box for group savings.
**Recurrent costs**			
Supervision and Feedback	Meetings with CHVs and health officials every 3 months (transportation, lunch, etc.)	10,214	The central program team provided regular supervision to the CHVs implementing the intervention as well as key stakeholders such as government and community health officials. CHVs participated in monthly meetings the first 3 months and then every 3 months for the remainder of the intervention. During these meetings, CHVs could provide feedback and ask questions and the program team could provide refresher training. We found these meetings to be essential for co-problem solving with CHVs and health officials.
Program management	Project manager and support staff	44,350	The intervention was supervised by a program team. Some of these team members were dedicated to activities related to the study (e.g., data collection and follow up of control subjects), however, we feel that the program requires significant management and coordination duties as well as program monitoring and evaluation to be successful. We have budgeted liberally for effort of 1 project manager, 1 project coordinator, and 1 monitoring and evaluation officer.

Total start up cost: $21,200
Total recurrent cost: $54,564
Total intervention cost: $75,764

A note on payment to CHVs
We did not include payment for CHVs since at the time of the study these individuals were not paid by the government in Kenya and were considered volunteers under the Community Health Strategy. We recognize that this could change in the future and that CHV salaries would represent a significant additional cost to the intervention. However, intervention activities that CHVs provided through the *Chamas* groups were considered part of their routine responsibilities for maternal and child health and their time and effort were designed to be consistent under these routine responsibilities. In other words, the CHVs who implemented the intervention were not full time and performed duties consistent with their role under the Kenya Community Health Strategy. Two CHVs were responsible for administering one

Chamas group through the intervention period which met for 1-1.5 hours twice per month. We hypothesize that by allowing CHVs to conduct maternal and child health visits in groups like *Chamas* that this makes their work more efficient than doing it by household.

Cost-Effectiveness of the *Chamas* Intervention

To assess the cost-effectiveness of the *Chamas* intervention, we calculated a simple cost per beneficiary and cost per outcome utilizing total intervention costs (start up and recurrent costs). These calculations likely represent significant over-estimates of costs as there are substantial cost savings that would be incurred by scaling this intervention at the County level and fully integrated into the public health system and Community Health Strategy. As this cost analysis was conducted as part of a randomized controlled trial, there were also costs associated with the implementation and evaluation of the intervention study that would not be incurred outside of the study setting. We are currently working on a larger cost analysis using data from our program implementation of *Chamas* which can be provided at a later date.

Cost per beneficiary

The total cost of the intervention was calculated at $75,764 and the total number of beneficiaries was 822 women, which equals a total **cost of $92 per beneficiary**. We also believe it is reasonable to include infants as beneficiaries as the intervention's primary outcomes were related to benefits to mothers and their infants. If mothers and infants are included as beneficiaries, this leads to an approximate **cost of $46 per beneficiary**.

Cost per outcome

The primary outcome of our intervention was increase in the likelihood of pregnant women delivering in a health facility. In multivariate regression modelling, we found that women who participated in our intervention was associated with a statistically significant 7.4% increase (80.9% vs. 73.0%) of delivering in a facility compared to women in the control group. Among the 807 women involved in the program that we had delivery information on, 653 women delivered in a health facility. Without the intervention program, we expect that only 589 of those women would have delivered in a health facility. The *Chamas* program led to 64 additional facility births among this cohort of 807 women at a **cost of $1,184 per additional health facility birth**.

Secondary outcomes targeted by this intervention were exclusive breastfeeding rates and infant immunizations, among others. We found that the intervention was associated with an 11.9% increase in exclusive breastfeeing and a 15.6% increase in infant immunization completion. This translated to 81 more women than usual choosing to exclusively breastfeed their child at a **cost of $935 per additional women exclusively breastfeeding**, and translated to 61 more children receiving all of their early child WHO recommended vaccinations at a **cost of $1,242 per additional fully vaccinated child**.

For this study, we looked at the outcome of the developmental screening questionnaire (DSQ). Forty-five children screen positive for at-risk development using the DSQ. Without the intervention program, we expect that 28 children would have had at-risk development. The Chamas program led to 11 fewer children having at-risk development, at a **cost of $6,888 per additional child without concerns for at-risk development.**
